# The Cut-Off Value of Serum Anti-Müllerian Hormone Levels for the Diagnosis of Turner Syndrome with Spontaneous Puberty

**DOI:** 10.1155/2023/6976389

**Published:** 2023-02-16

**Authors:** Jin Wang, Tian Lan, Xiang Dai, Luhong Yang, Xijiang Hu, Hui Yao

**Affiliations:** Wuhan Children's Hospital (Wuhan Maternal and Child Healthcare Hospital), Tongji Medical College, Huazhong University of Science and Technology, Wuhan 430015, China

## Abstract

**Objective:**

Preservation of fertility in Turner syndrome (TS) patients may be feasible through cryopreservation of ovarian tissue before follicles begin to disappear. Anti-Müllerian hormone (AMH) is said to be a predictive factor of spontaneous pubertal development in TS. We aimed to determine the cut-off values of AMH for the diagnosis of TS girls with spontaneous puberty. *Design and methods*: A total of 95 TS patients between 4 and 17 years were evaluated at the Department of Pediatric Genetic Metabolism and Endocrinology from July 2017 to March 2022. Serum AMH, follicle-stimulating hormone (FSH), and luteinizing hormone (LH) levels were analyzed according to age, karyotype, pubertal development, and ultrasound ovarian visualization. Receiver-operating characteristic (ROC) curve analyzes were used to test the utility of AMH for the diagnosis of TS girls with spontaneous puberty.

**Results:**

One-fourth of TS girls aged 8–17 years had spontaneous breast development, with the ratios as follows: 45, X (6/28, 21.4%), mosaicism (7/12, 58.3%), and mosaicism with structural X chromosome abnormalities (SCA) (2/13, 15.4%), SCA (1/13, 7.7%), and Y chromosome (1/3, 33.3%). The AMH cut-off value for the prediction of spontaneous puberty in TS patients was 0.07 ng/ml, with sensitivity and specificity both at 88%. FSH, LH levels, and Karyotypes could not be considered as markers of spontaneous puberty in TS (*P* > 0.05). A strong relationship was observed between serum AMH levels and spontaneous puberty or ultrasound bilateral ovarian visualization.

**Conclusions:**

The AMH cut-off value for the prediction of spontaneous puberty in TS girls aged 8–17 years was 0.07 ng/ml, with sensitivity and specificity both at 88%. However, spontaneous puberty in these patients is not predictable based on karyotype or FSH or LH levels.

## 1. Introduction

Turner syndrome (TS) is one of the most common genetic disorders, with an incidence of 1 : 1200–1 : 2500 live-born girls [1, 2]. It is caused by the complete or partial loss or complex rearrangement of one X chromosome [3]. Its main clinical manifestations include hypergonadotropic hypogonadism due to gonadal dysgenesis, which leads to premature ovarian insufficiency (POI) and subsequent infertility [4–6]. According to the literature [7, 8], approximately 30% of TS patients have spontaneous pubertal development, 5%–10% have regular menstrual cycles, and ∼2% experience spontaneous pregnancies. The advancement of reproductive medicine provides the possibility of salvaging fertility in patients with TS whose oocytes or ovarian tissues are collected and preserved before follicles begin to disappear. Premature ovarian failure usually occurs before the reproductive age in patients with TS. Purushothaman et al. [9] recommended that follicle retrieval should be done in TS patients at 12–13 years old. Therefore, biochemical markers reflecting the ovarian reserve in childhood TS patients as early as possible are needed.

The TS karyotype has been reported to be highly associated with ovarian status in TS patients [10]; however, other studies have shown that there is no correlation between karyotype and ovarian status. It was reported that a small number of TS patients with karyotype 45, X can experience spontaneous development, menstrual regularity, and even natural pregnancy [11, 12]. Accurately predicting ovarian reserve and fertility potential-based only on chromosome karyotype is difficult. In addition, studies have suggested that the ovaries of all TS patients with normal fertility can be visualized by transabdominal ultrasound, whereas ovaries of TS patients with subfertility cannot be visualized [9]. It is still unclear whether the ovarian reserve function of TS patients with bilateral ovarian development is better than that of patients with ovarian dysplasia or unilateral ovarian development.

AMH has been reported as a predictive factor of spontaneous pubertal development in TS [13]. To date, the cut-off value of AMH in TS patients for predicting spontaneous pubertal development has not yet been established. Circulating AMH reflects the number of primordial follicles and is therefore predictive of reproductive lifespan [14, 15]. In healthy adult women, serum AMH levels are strongly correlated with antral follicle counting (AFC) [16], and the decline in serum AMH levels precedes the changes in traditional markers of ovarian reserve, such as FSH, inhibin B, and E2 [17, 18]. Serum AMH concentrations are shown to be a valuable marker of ovarian function in girls, adolescents, and adult women with TS.

The aim of this study was to determine the cut-off value of AMH levels for the diagnosis of TS girls aged 8–17 years with spontaneous puberty, correlating the AMH value with pubertal development and ovary visualization on ultrasound scans.

## 2. Materials and Methods

### 2.1. Participants

A total of 105 TS patients were recruited from the Genetic Metabolism and Endocrinology, Wuhan Children's Hospital Tongji Medical College, Huazhong University of Science and Technology from July 2017 to March 2022. Ten of the 105 patients were excluded from the study because of incomplete data, and 95 patients were finally included in the study. The diagnosis of TS was based on a peripheral blood leucocyte karyotype analysis. The mean age of all patients was 10 ± 4 years (4–17 years). TS patients were divided into two age groups: 4–7 years (26/95, 27.4%) and 8–17 years (69/95, 72.6%). Clinical data regarding the age of TS, chromosomal karyotype, spontaneous puberty, and Tanner pubertal staging were collected. This study was approved by the ethics committee of Wuhan Children's Hospital. Guardians of all recruited volunteers provided informed consent for the analysis of data for publication. The data were supplied anonymously. According to the standards of Tanner staging of breast development [19], of the 95 patients studied, 78 were in Tanner stage 1, 8 in Tanner stage 2, 7 in Tanner stage 3, and 2 in Tanner stage 4.

Analyzes for spontaneous puberty included TS patients aged 8 years and above who had no sex hormone replacement therapy. Breast development at Tanner stage B2 or more was taken as the definition for spontaneous pubertal development [20]. TS patients aged 4–7 years with no spontaneous pubertal development were in Tanner stage 1. According to the standards of Tanner staging of breast development, TS patients aged 8–17 years were further classified into two age groups: 8–12 years and 13–17 years. Of the 12 TS patients in the 8–12 years group, 7 were in Tanner stage 2, and 5 in Tanner stage 3. Of the 5 TS patients in the 13-17 years group, 1 was in Tanner stage 2, 2 in Tanner stage 3, and 2 in Tanner stage 4. As a control population, we enrolled healthy girls aged 4–17 years. The exclusion criteria were as follows. Girls with Turner syndrome, a suspected disorder of sexual development, PCOS, premature ovarian insufficiency, endocrine disease, inherited metabolic disease, liver malfunction, kidney malfunction, kidney disease, circulatory system diseases, acute or chronic infections, prescribed or nonprescribed medications, and girls who had undergone surgery were excluded from the study [21].

### 2.2. Karyotypes

The diagnosis of TS was confirmed by karyotyping using routine G-banding, including the counting of at least 10 metaphases, three of which were fully analyzed. Chromosome polymorphisms were recorded. All the karyotypes in the present study were validated by the same clinician. TS patients were divided into five groups according to karyotype: 45, X, mosaicism, mosaicism with SCA, SCA, and Y chromosome material.

### 2.3. Hormone Assays and Ultrasonography

All samples were withdrawn from the antecubital vein, clotted, centrifuged, and analyzed. Serum AMH levels were determined using the Beckman Access 2 automated chemiluminescence immunoassay within the lowest limit of quantitation (0.01 ng/mL). Quality control (QC) was used to monitor the precision of the instrument. The assay was evaluated for intra- and inter-assay precisions. To estimate the intra-assay coefficients of variation (CV), three known QC materials with AMH concentrations of 1.0, 5.0, and 15.0 ng/mL were measured 10 times in a single assay. To evaluate inter-assay CV, three AMH concentrations (1.0, 5.0, and 15.0 ng/mL) were measured on 10 consecutive days. The mean, standard deviation (SD), and CV values were calculated. Intra- and inter-assay coefficients of variation for serum AMH were 1.97–2.86% and 2.24–3.85%, respectively. LH and FSH levels were measured using the Beckman immunochemiluminometric assay. Intra- and inter-assay coefficients of variation were 3.6–4.7% for FSH and 3.4–4.8% for LH.

Ultrasonographic visualization of the ovaries with transabdominal pelvic ultrasound examinations was performed using a 5-MHz convex transducer (Siemens Acuson Antares 5.0, Acuson Sequoia). A detectable hypoechogenic but unmeasurable structure without follicles could not be accurately distinguished from streak gonads and was not considered a true ovary. Patients were divided into ovarian dysplasia or unilateral ovarian visualization and bilateral ovarian visualization groups.

### 2.4. Statistics

SPSS version 25.0 (IBM, Armonk, NY, USA) was used to perform the data analysis. The Kolmogorov–Smirnov test was used to determine the data distribution. Means and SDs were used to express normally distributed data, whereas non-normally distributed data were expressed as medians with 25^th^–75^th^ percentiles. The Kruskal–Wallis H test was used to determine differences in AMH levels between the five karyotype groups of TS patients. The Kruskal–Wallis H test was used to determine differences in FSH and LH levels between the karyotype groups of TS patients. In TS patients, a receiver operating characteristic (ROC) curve was used to determine the cut-off value between nonspontaneous and spontaneous puberty in terms of serum AMH. The correlations between serum AMH levels and spontaneous puberty or ovarian visualization were determined using binary logistic regression.

## 3. Results

### 3.1. Description of TS Patients

Descriptions of TS patients according to karyotype are presented in Table 1. TS patients were divided into five groups according to karyotype: 45, X (*n* = 41, 43.2%), mosaicism (*n* = 16, 16.8%), and mosaicism with SCA (*n* = 17, 17.9%), SCA (*n* = 16, 16.8%), and those with Y chromosome material (*n* = 5, 5.3%).

### 3.2. Descriptive Characteristics of Five Karyotype Groups of TS Patients

**Figure 1:**
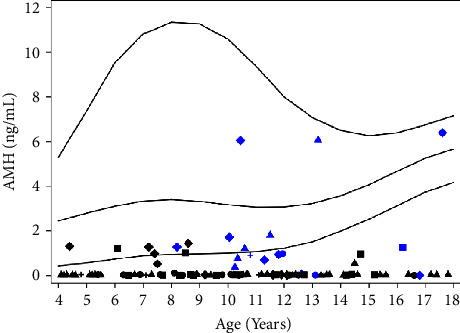
Serum AMH levels in TS adolescent girls with spontaneous puberty. The reference range is based on healthy Chinese girls. Lines represent the median, the 2.5^th^ percentile, and the 97.5^th^ percentile. Serum concentrations of AMH (ng/ml) of 95 TS patients are plotted versus age. Triangles: 45, X; diamond: mosaicism; squares: mosaicism with SCA; circle: SCA; cruciform: with Y chromosome; black: TS patients with nonspontaneous puberty; blue: TS patients with spontaneous puberty. pmol/L = ng/mL *∗* 7.14.

In patients aged 8–17 years, the ratios of those with spontaneous pubertal development according to karyotype were as follows: 45, X (6/28, 21.4%), mosaicism (7/12, 58.3%), mosaicism with SCA (2/13, 15.4%), SCA (1/13, 7.7%), and Y chromosome material (1/3, 33.3%) (Table 2). In 45, X/46, XX mosaicism, the ratio of spontaneous puberty reached 60%. The ratio of TS patients with spontaneous pubertal development was lower with SCA than that of those with other karyotypes. A scatter plot of AMH levels in TS patients with spontaneous puberty or nonspontaneous puberty is shown in Figure 1. Karyotypes are not correlated with spontaneous pubertal development of the TS girls, and cannot be a predictor of spontaneous pubertal development of the TS girls. In patients aged 4–7 years, the AMH levels showed a significant difference between the five karyotype groups. The median value of the AMH level in the mosaicism group was 1.13 ng/ml, which was significantly higher than that in the other groups. In patients aged 8–17 years, AMH levels showed no significant difference among the five karyotype groups. In patients aged 4–7 and 8–17 years, there were no differences in FSH and LH levels among the five karyotype groups (Table 2).

### 3.3. AMH, FSH, and LH Levels and Spontaneous Puberty in TS Patients

A fourth of pubertal TS patients had spontaneous breast development. At ages 8–12 and 13–17 years, regardless of karyotype, TS patients with spontaneous puberty had higher serum AMH levels than those with nonspontaneous puberty, which were lower than those in the control group aged 8–12 and 13–17 years (Table 3). In the same way, FSH and LH levels show a statistically significant difference among patients with spontaneous, nonspontaneous puberty, and control groups. Noteworthy, the AMH level in the TS patients aged 8–12 with spontaneous puberty group is 1.10(0.74∼1.52), and that in the TS patients aged 13–17 with spontaneous puberty group is 1.27(0.02∼6.07). These results do not show a statistically significant difference (Figure 1 and Table 3), indicating that the AMH level in TS patients with spontaneous puberty development does not show a significant difference according to age, in contrast to the fluctuation of the AMH level in healthy persons according to age.

### 3.4. AMH as a Marker of Spontaneous Puberty in TS Patients

**Figure 2:**
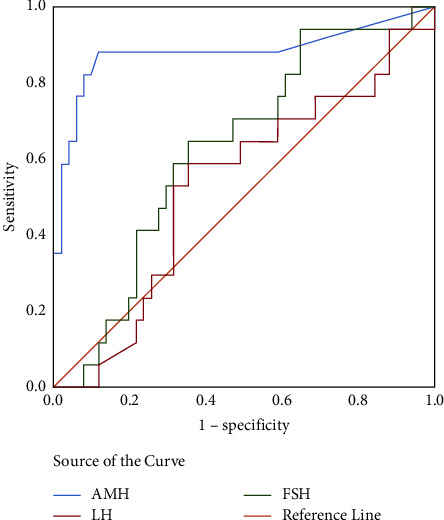
ROC curve showing cutoff values of AMH for predicting spontaneous puberty.

We performed ROC analyzes of TS patients aged 8–17 years with spontaneous puberty development to determine the cut-off value for AMH levels. The best cut-off value for AMH as a marker of spontaneous puberty in TS patients was 0.07 ng/ml. Both the sensitivity and specificity of this test were 88% (Figure 2). FSH and LH levels could not be considered as markers of spontaneous puberty in TS patients (*P* > 0.05) (Table 4). AMH level was associated with spontaneous puberty (OR: 23.49; 95% CI 4.89–112.85; *P*  < 0.001) in TS patients.

### 3.5. Correlations between AMH Levels in TS Patients and Bilateral Ovarian Visualization

At ages 4–7 and 8–17 years, the AMH level was associated with the visualization of bilateral ovaries on ultrasonography scans (OR: 12.64; 95% CI 1.25–128.36; *P*=0.032 and OR: 4.31; 95% CI 1.47–12.64; *P*=0.008, respectively).

## 4. Discussion

This was a cross-sectional study of 95 TS patients. Although the main clinical feature of most TS patients is premature ovarian insufficiency [13, 22], some patients still experience spontaneous puberty [23]. Serum AMH levels have been shown to reflect ovarian reserves in adult women with TS [24]. Our study is to establish the cut-off value of AMH in TS girls for predicting spontaneous puberty regardless of karyotype. Our ROC curve showed a cutoff point of 0.07 ng/ml for AMH levels separating between spontaneous puberty and nonspontaneous puberty in TS girls aged 8–17 years, with both sensitivity and specificity at 88% each. On the contrary, FSH and LH levels cannot be used as markers of spontaneous puberty in TS patients (*P* > 0.05). These results are consistent with those reported by Hankus et al. [25], who argued that FSH has poor sensitivity and specificity for predicting the spontaneous development of TS. AMH is a good predictor of spontaneous puberty in TS patients. Therefore, we performed ROC analyzes of TS patients aged 8–17 years with spontaneous puberty development to determine the cut-off value for AMH levels.

Our study showed that spontaneous puberty in TS patients was not predictable based on karyotype. In 45, X/46, and XX mosaicism, the ratio of spontaneous puberty reached 60%. The ratio of TS patients with spontaneous pubertal development was lower with SCA than that of those with other karyotypes. In this study, karyotype groups (45, X, mosaicism, and mosaicism with SCA, SCA, and Y chromosome materials) are different from previous karyotype groups [26]. The proportion of spontaneous development among these five karyotype groups showed differences [10]. Hankus et al. [25] believed that although spontaneous puberty is more frequent in non-45, X patients, the karyotypes cannot be used to predict it. Our study also showed that karyotypes are not correlated with spontaneous pubertal development of the TS patients and cannot be a predictor of spontaneous pubertal development of the TS patients.

In the present study, AMH levels were positively correlated with spontaneous puberty. According to Visser's report [27], the ELISA method was used to detect AMH concentrations of 0.037–5 ng/ml. When the AMH concentration was lower than 0.037 ng/ml, it could not be determined. The overall AMH was only measurable in 21.9% of TS subjects in Visser's report. Visser's study found that a strong relationship between the measurable serum AMH concentrations and signs of spontaneous puberty, such as breast development. These results were consistent with our findings. But the chemiluminescence method [28–30] used in our study to detect AMH levels is different from the ELISA method in the Visser's study. In our study, chemiluminescence was used to detect AMH levels in 95 girls aged between 4 and 17 years. The lowest detectable concentration was 0.01 ng/ml. AMH concentrations were determined in all patients. The results showed that AMH levels were positively correlated with spontaneous puberty. This suggests that AMH level is a proper indicator of adolescent development in patients with TS. Furthermore, our study showed that the AMH levels were associated with bilateral ovarian visualization. Therefore, AMH levels might play an important role in evaluating ovarian function in TS girls.

We analyzed the relationship between serum AMH levels and different TS karyotypes. Our study showed significant differences in AMH levels among the five TS karyotype groups in TS patients aged 4–7 years. In mosaicism, the median AMH was 1.13 ng/ml, which was significantly higher than that in the other groups. There was no significant difference in AMH levels among the five karyotypes in patients aged 8–17 years. We inferred that, at age 4–7 years, the ovarian reserve function of TS patients with mosaicism was better than that of patients with other karyotypes. However, as the age of these patients with mosaicism increases, their ovarian reserve function decreases. In addition, there were also no differences in FSH and LH levels among patients with the five karyotypes. On the other hand, our study had limitations. First, we have a limited number of cases. Second, we did not analyze the ratio of TS patients with spontaneous menstruation and its relationship with AMH levels. This is especially important given that the average age of our patients was approximately 10 years, while the average time of spontaneous menstruation was around 12 years [31, 32]. Therefore, we will follow all TS patients to further study the ratio of spontaneous menstruation, focusing on whether AMH or other hormones can predict spontaneous menstruation of TS patients, and the correlations between the AMH levels and the TS patients with spontaneous pregnancy or POI in order to provide a theoretical support for the fertility evaluation of children with TS.

In conclusion, the AMH cut-off value for the prediction of spontaneous puberty in TS girls aged 8–17 years was 0.07 ng/ml, with sensitivity and specificity both at 88%. However, the spontaneous puberty in these patients is not predictable based on karyotype, FSH, or LH levels. AMH level as a specific marker for predicting spontaneous puberty helps screen the TS girls with puberty development as early as possible. This can provide the early support and counseling for the fertility-related problems that TS patient's experience.

## Figures and Tables

**Table 1:** Chromosomal karyotypes of 95 TS patients (age 4–17 years).

Karyotype	*n*	Percentage (%)
45, X	41	43.2

Mosaicism	16	16.8
45, X/46, XX	10	
45, X/46, XXX	2	
45, X/47, XXX	3	
45, X/46, XX/47, XXX	1	

Mosaicism with SCA	17	17.9
45, X/46, X, i (X) (q10)	6	
45, X/46, XX, +Mar	3	
45, X/46, X, r (X)	2	
45, X/46, X, del (X) (p10)	1	
45, X/46, X, del (X) (p11.2)	1	
45, X/46, X, i (Xq)	2	
45, X/46, X, i (q10)/47, X, i (q10)	2	

SCA	16	16.8
46, X, i (X) (q10)	10	
46, X, del (X) (q21)	1	
46, X, del (X) (q22)	1	
46, X, del (X) (p10)	1	
46, X, del (X) (q113)	1	
46, XX, del (X) (p11)	1	
46, X, add (X) (p21)	1	

Y chromosome material	5	5.3
45, X/46XY	2	
45, X/45, XY	1	
45, X/47, XYY	1	
45, X/46XY/47, XYY	1	

SCA: structural X chromosome abnormalities; Mosaicism with SCA: mosaicism with structural X chromosome abnormalities.

**Table 2:** Descriptive characteristics of five karyotype groups in TS patients.

Variables	45, X	Mosaicism	Mosaicism with SCA	SCA	Y chromosome material	*P* values
4–7 years						
*N*	13	4	4	3	2	
Age	4.92 ± 1.17	6.25 ± 1.50	6.25 ± 0.50	6.33 ± 0.58	5.50 ± 2.12	0.147
AMH (ng/ml)	0.02 (0.02–0.03)	1.13 (0.75–1.30)	0.04 (0.04–0.05)	0.02 (0.02–0.62)	0.03 (0.02–0.04)	0.009
LH (mIU/ml)	0.07 (0.07–0.18)	0.05 (0.03–0.07)	0.14 (0.09–0.15)	0.07 (0.07–0.08)	0.08 (0.07–0.09)	0.272
FSH (mIU/ml)	12.60 (7.23–41.14)	2.00 (1.07–5.99)	16.00 (13.66–24.36)	8.04 (5.00–9.06)	9.10 (3.65–14.55)	0.07
Ovarian ultrasonography						0.007
Ovarian dysplasia or unilateral ovarian visualization	13 (100%)	1 (25%)	2 (50%)	2 (66.7%)	1 (50%)	
Bilateral ovarian visualization	0 (0%)	3 (75%)	2 (50%)	1 (33.3%)	1 (50%)	
8∼17						
*N*	28	12	13	13	3	
Age	12.39 ± 2.56	11.18 ± 2.93	11.00 ± 2.35	11.31 ± 3.01	10.67 ± 2.08	0.424
AMH (ng/ml)	0.03 (0.02–0.07)	0.70 (0.03–1.59)	0.03 (0.02–0.04)	0.03 (0.02–0.05)	0.06 (0.02–0.10)	0.346
LH (mIU/ml)	11.21 (0.82–17.35)	1.23 (0.20–27.81)	7.89 (0.23–26.39)	15.89 (0.48–19.04)	2.84 (1.54–15.65)	0.983
FSH (mIU/ml)	55.18 (11.79–90.36)	17.02 (2.92–82.97)	64.09 (9.29–105.33)	58.57 (20.50–94.18)	23.90 (16.25–58.87)	0.606
Ovarian ultrasonography						0.619
Ovarian dysplasia or unilateral ovarian visualization	18 (64.3%)	5 (41.7%)	9 (69.2%)	10 (76.9%)	2 (66.7%)	
Bilateral ovarian visualization	10 (35.7%)	7 (58.3%)	4 (30.8%)	3 (23.1%)	1 (33.3%)	
Spontaneous puberty						0.032
No	22 (78.6%)	5 (41.7%)	11 (84.6%)	12 (92.3%)	2 (66.7%)	
Yes	6 (21.4%)	7 (58.3%)	2 (15.4%)	1 (7.7%)	1 (33.3%)	

Data are expressed as means ± SDs or as medians (25^th^–75^th^ percentiles). FSH; follicle-stimulating hormone; LH: luteinizing hormone; AMH: Anti-Müllerian hormone. pmol/L = ng/mL ∗ 7.14.

**Table 3:** AMH, LH, and FSH levels in TS patients with spontaneous puberty compared to those with nonspontaneous puberty and control.

	8–12 years				13–17 years			
	Control	Nonspontaneous puberty	Spontaneous puberty	*p* values	Control	Nonspontaneous puberty	Spontaneous puberty	*p* values
AMH (ng/ml)	3.02 (2.01∼4.48)	0.03 (0.02∼0.04)	1.10 (0.74∼1.52)	<0.001	2.93 (2.34∼4.31)	0.03 (0.02∼0.03)	1.27 (0.02∼6.07)	<0.001
FSH (mIU/ml)	3.44 (2.11∼5.22)	31.06 (4.91∼64.15)	18.95 (4.38∼28.80)	<0.001	5.13 (3.22∼7.79)	93.84 (69.39∼99.49)	65.71 (57.22∼77.97)	<0.001
LH (mIU/ml)	0.29 (0.08∼1.27)	1.99 (0.07∼15.34)	1.11 (0.20∼2.71)	0.11	4.90 (1.86∼8.05)	20.60 (17.08∼26.81)	26.39 (19.04∼31.86)	0.02

Results are expressed as medians with the 25^th^–75^th^ percentiles. pmol/L = ng/mL*∗*7.14.

**Table 4:** ROC curve values for predicting spontaneous puberty in TS aged 8–17 years.

Variables	Area	Std	*P* values	95%CI
AMH	0.882	0.063	<0.001	0.759∼1.000
LH	0.531	0.081	0.702	0.373∼0.690
FSH	0.629	0.073	0.114	0.485∼0.772

## Data Availability

Some or all datasets generated during and/or analyzed during the current study are not publicly available but are available from the corresponding author on reasonable request.
